# Identification of Selective Agonists and Antagonists to G Protein-Activated Inwardly Rectifying Potassium Channels: Candidate Medicines for Drug Dependence and Pain

**DOI:** 10.2174/157015911795017227

**Published:** 2011-03

**Authors:** D Nishizawa, N Gajya, K Ikeda

**Affiliations:** 1 Division of Psychobiology, Tokyo Institute of Psychiatry, Tokyo; 2 Discovery Biology-1, Discovery Biology Research, Global Research & Development, Nagoya Laboratories, Pfizer Japan Inc, Nagoya, Japan

**Keywords:** G protein-activated inwardly rectifying K^+^ (GIRK, Kir3) channels, Kir channel, agonist, antagonist, Pfizer compounds, *Xenopus* oocyte.

## Abstract

G protein-activated inwardly rectifying K^+^ (GIRK) channels have been known to play a key role in the rewarding and analgesic effects of opioids. To identify potent agonists and antagonists to GIRK channels, we examined various compounds for their ability to activate or inhibit GIRK channels. A total of 503 possible compounds with low molecular weight were selected from a list of fluoxetine derivatives at Pfizer Japan Inc. We screened these compounds by a *Xenopus* oocyte expression system. GIRK1/2 and GIRK1/4 heteromeric channels were expressed on *Xenopus laevis* oocytes at Stage V or VI. A mouse IRK2 channel, which is another member of inwardly rectifying potassium channels with similarity to GIRK channels, was expressed on the oocytes to examine the selectivity of the identified compounds to GIRK channels. For electrophysiological analyses, a two-electrode voltage clamp method was used. Among the 503 compounds tested, one compound and three compounds were identified as the most effective agonist and antagonists, respectively. All of these compounds induced only negligible current responses in the oocytes expressing the IRK2 channel, suggesting that these compounds were selective to GIRK channels. These effective and GIRK-selective compounds may be useful possible therapeutics for drug dependence and pain.

## INTRODUCTION

G protein-activated inwardly rectifying K^+^ (GIRK) channels, also named as Kir3 channels, are members of the inwardly rectifying potassium channel family. GIRK channels are activated by several G_i/o_ protein-coupled receptors, such as opioid receptors, which causes hyperpolarization of the neurons involved and thus leads to inhibitory regulation. GIRK channels are expressed in many tissues with different subunit compositions [1-3]. In the heart, the GIRK4 subunit is abundantly expressed as a homomultimer or heteromultimer with GIRK1 and is involved in heart rate regulation [4, 5]. In the central nervous system, GIRK channels are mainly expressed as a GIRK1/2 heteromultimer in most regions and as a GIRK2 homomultimer in the substantia nigra and ventral tegmental area. GIRK channels play a key role in analgesia [6], as demonstrated in studies using GIRK channel subunit knockout mice [7-11]. Further, mice lacking the GIRK2 or GIRK3 subunit show decreased cocaine self-administration, suggesting decreased reinforcing effects of cocaine in these mice [12] and hence the involvement of GIRK channels in its rewarding effects.

Therefore, GIRK channel inhibitors may be possible candidates as therapeutic drugs to treat substance dependence. Drugs that selectively open GIRK channels may be expected to exhibit analgesic effects without impacting opioidergic intracellular signaling pathways and G_i/o_ proteins and thus have fewer side effects. It has been known that various compounds inhibit GIRK channels [13-17], but only a few have thus far been shown to activate the GIRK channel [18-20]. To identify more potent GIRK channel agonists and antagonists, we examined the ability of various compounds to activate or inhibit GIRK channels.

## METHODS

### Compounds

To search for selective GIRK channel agonists and antagonists, a total of 503 possible compounds with low molecular weight were selected from a list of fluoxetine derivatives at Pfizer Japan Inc. The specific names and detailed properties of each compound are not available to the public. For convenience, the compounds were numbered from PF 1 to PF 503. All drugs were dissolved in dimethyl sulfoxide (DMSO).

### Electrophysiological Analysis

To screen the PF compounds, a *Xenopus* oocyte expression system was utilized based on a previous report [21]. In this system, murine GIRK1 (Kir3.1), GIRK2 (Kir3.2), and GIRK4 (Kir3.4) subunits were expressed as heteromeric channels of GIRK1/2 and GIRK1/4 in *Xenopus* *laevis* oocytes at Stage V or VI by coinjection of the cRNAs of mouse GIRK1 and GIRK2 subunits, and GIRK1 and GIRK4 subunits, respectively. The murine IRK2 (Kir2.1) channel, which is a member of another inwardly rectifying potassium channel family with similarity to the GIRK channel family, was expressed in the oocytes to examine the selectivity of the identified compounds to GIRK channels. For electrophysiological analyses, a two-electrode voltage clamp (GeneClamp500, Axon Instruments) method was used with the membrane potential kept at -70 mV. A high potassium solution (96 mM KCl, 2 mM NaCl, 1 mM MgCl_2_, 1.5mM CaCl_2_, 5 mM HEPES) served as perfusion solution. Ethanol (100 mM) and BaCl_2_ (2 mM) were used as positive controls for agonist and antagonist, respectively, and DMSO was used as a negative control. Oocytes without cRNA injection served as controls.

### Assay Procedure

The procedure of the assay consisted of three steps. In the first step, among the total of 503 PF compounds, every four compounds were mixed together and dissolved in the high potassium solution to yield a solution containing each compound at 10 μM. Then the total of 126 solutions of pooled PF compounds were applied to the oocytes expressing the GIRK1/2 channel (*n* = 2), GIRK1/4 channel (*n* = 2), and oocyte controls (*n* = 2) without GIRK channel expression. After the first screening step, several pools of compounds were selected based on the following criteria: (i) stronger agonistic or antagonistic effect on GIRK channels, (ii) similar responses between the two oocytes tested, and (iii) substantial difference in the effect of activation or inhibition between GIRK1/2 and GIRK1/4 channels.

In the second step, PF compounds in the selected pools (10 μM) were separately applied to the oocytes expressing the GIRK1/2 channel (*n* = 2), GIRK1/4 channel (*n* = 2), and oocyte controls (*n* = 2) without GIRK channel expression. Several compounds were selected based on the same criteria as those in the first step, in which their magnitude of inhibition/activation and the selectivity for the GIRK1/2 or GIRK1/4 channel were considered.

In the third step, each selected PF compound was applied to the oocytes expressing the GIRK1/2 channel (*n* = 5) and GIRK1/4 channel (*n* = 5) at various concentrations to examine concentration-response relationships. Selectivity of the compounds for GIRK channels was tested by applying the compounds to the oocytes expressing IRK2 (*n* = 2) and oocyte controls (*n* = 2) without GIRK channel expression. Data were fitted to a standard regression equation by using KaleidaGraph 3.5J (HULINKS, Inc.) for analysis of concentration-response relationships.

### Statistical Analyses

The current responses to PF compounds were normalized by those to ethanol or BaCl_2_, which was applied to each oocyte like PF compounds. For statistical analyses, two-way analysis of variance (ANOVA) or Student’s *t*-test was performed with the significance level set at *P* < 0.05. SPSS v.12.0J for Windows (LEAD Technologies, Inc.) was used for analyses.

## RESULTS

**Fig. (1):**
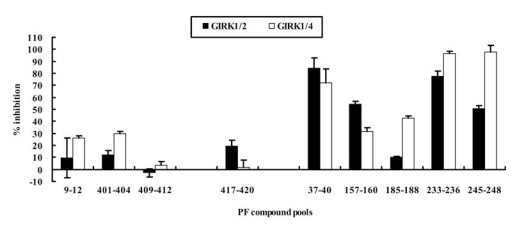
**Current Responses Induced by the Selected PF Compound Pools in the First Screening Step.** Normalized current responses to the pools of PF compounds by the response to BaCl_2_. PF 9 – PF 12, PF 401 – PF 404, and PF 409 – PF 412 pools were selected as candidate agonists, and PF 37 – PF 40, PF 157 – PF 160, PF 185 – PF 188, PF 233 – PF 236, and PF 245 – PF 248 pools were selected as candidate antagonists. The PF 417 – PF 420 pool was selected as a candidate agonist or antagonist in the first screening step.

In the first screening step, some pools of PF compounds showed agonistic effects on GIRK channels while most others showed antagonistic effects on GIRK1/2 and GIRK1/4 channels with various efficacies. All of the pools of PF compounds induced negligible responses in the oocytes without cRNA injection (data not shown), suggesting the current responses by PF compounds were caused by exogenously expressed GIRK channels. Based on the criteria described above, PF 9 – PF 12, PF 401 – PF 404, and PF 409 – PF 412 pools were selected as candidate agonists with relatively low percentage inhibition of GIRK currents compared to BaCl_2_ responses, while PF 37 – PF 40, PF 157 – PF 160, PF 185 – PF 188, PF 233 – PF 236, and PF 245 – PF 248 pools were selected as candidate antagonists with relatively high percentage inhibition of GIRK currents (Fig. **1**). In addition, the PF 417 – PF 420 pool was selected as candidate agonist or antagonist because it induced moderate inhibition of GIRK1/2 channels and almost no effect on GIRK1/4 channels (Fig. **1**).

**Fig. (2):**
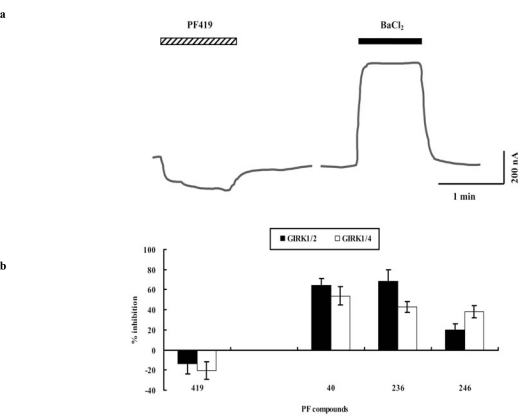
**Candidate Agonist and Antagonists Identified in the Second Screening Step.** PF 419 was selected as a candidate agonist, and PF 40, PF 236, and PF 246 were selected as candidate antagonists in the second screening step. **a**. Traces of typical current responses to PF 419 (30 µM) and BaCl_2_ (2 mM) in the oocyte expressing the GIRK1/4 channel. The striped and filled bars represent the duration of the application of PF 419 and BaCl_2_, respectively. **b**. The normalized current responses to the selected PF compounds by the response to BaCl_2_.

In the second screening step, the PF compounds in the nine pools selected above were separately applied to the oocytes. While most of these individual PF compounds showed antagonistic effects or almost no effect on GIRK channels, only PF 419 showed an apparent agonistic effect on both GIRK1/2 and GIRK1/4 cannels (Fig. **2a**, **b**). Therefore, PF 419 was selected as a candidate agonist. In addition, PF 40, PF 236, and PF 246, which induced comparatively higher percentage inhibition of GIRK currents, were selected as candidate antagonists (Fig. **2b**).

**Fig. (3):**
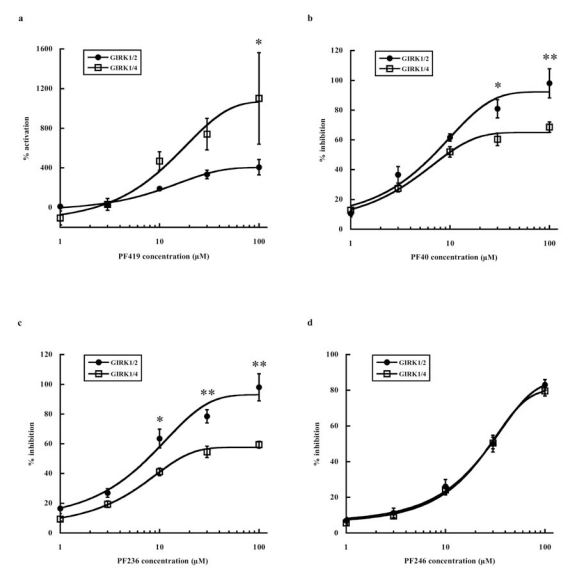
**Concentration-Response Relationships of the Identified Agonist and Antagonists to GIRK Channels. a.** Current response was normalized by the response to ethanol (100 mM). **b-d**. Current responses were normalized by the response to BaCl_2_ (2 mM). *P < 0.005 between GIRK1/2 and GIRK1/4. **P < 0.001 between GIRK1/2 and GIRK1/4.

In the third screening step, the four PF compounds selected above (PF 419, PF 40, PF 236, PF 246) were applied to the oocytes expressing the GIRK1/2 channel (*n* = 5) and GIRK1/4 channel (*n* = 5) at various concentrations. Considering the results in our preliminary experiments testing the effect of each compound at 0.1, 1, and 10 μM (data not shown), the concentrations of each compound to examine the concentration-response relationships were set at 1, 3, 10, 30, and 100 μM. Fig. (**3**) represents the concentration-response relationships for each PF compound. PF 419 activated GIRK currents dose-dependently, whereas PF 40, PF 236, and PF 246 inhibited them dose-dependently. Two-way ANOVA revealed that there were significant main effects of the concentrations of PF compounds on the current responses to PF 419 (F_4,40_ = 8.606, *P* < 0.001), PF 40 (F_4,40_ = 75.475, *P* < 0.001), PF 236 (F_4,40_ = 80.160, *P* < 0.001), and PF 246 (F_4,40_ = 227.702, *P* < 0.001). There were significant main effects of the GIRK subunit compositions on the current responses in PF 419 (F_1,40_ = 6.078, *P* = 0.018), PF 40 (F_1,40_ = 19.865, *P* < 0.001), and PF 236 (F_1,40_ = 50.590, *P* < 0.001). Significant interactions were observed between the concentrations and GIRK subunit compositions in PF 40 (F_4,40_ = 3.252, *P* = 0.021) and PF 236 (F_4,40_ = 4.371, *P* = 0.005). *Post hoc* analysis revealed a significant difference between the GIRK1/2 and GIRK1/4 channels at 100 μM (*P* = 0.004) in PF 419, at 30 μM (*P* = 0.004) and 100 μM (*P* < 0.001) in PF 40, and at 10 μM (*P* = 0.001), 30 μM (*P* < 0.001), and 100 μM (*P* < 0.001) in PF 236 (Fig. **3**). Inhibition concentration (IC_50_) values were calculated for PF 40, PF 236, and PF 246 (Table **1**). In addition, we examined the selectivity of these compounds for GIRK channels by applying them to oocytes expressing the IRK2 channel (*n* = 2). All four compounds showed almost no or negligible effects on IRK2 channels (data not shown), suggesting that these compounds were selective for GIRK channels.

## DISCUSSION

In the first step of the screening process, most pools of PF compounds showed apparent antagonistic effects and a few showed agonistic effects on GIRK channels, possibly because the PF compounds used in the present assay were selected from the fluoxetine derivatives that are known to inhibit GIRK channels [22]. However, several pools of PF compounds exhibited almost no effects on GIRK channels. It might be possible that agonists and antagonists were combined in a pool which effectively negated their possible actions on GIRK channels. Therefore, if different combinations of PF compounds had been pooled, different agonists and antagonists might have been identified after the overall screening procedure.

As shown in Table **1**, the IC_50_ values for PF 40, PF 236, and PF 246 calculated in the third screening step ranged from 5.98 to 31.2. These were comparable to, or lower than, those of various antipsychotic drugs [14-16], indicating they could be more potent antagonists than therapeutic drugs currently available. In addition, the agonistic effect of PF 419 at 10 μM or higher was several times that of ethanol (100 mM), a well-known GIRK agonist [18, 19], indicating that PF 419 is a very potent GIRK agonist. In a comparison between GIRK1/2 and GIRK1/4, significant differences were observed in PF 419, PF 40, and PF 236 (Fig. **3**), suggesting that PF 40 and PF 236 are antagonists relatively selective to GIRK1/2 and PF 419 is an agonist relatively selective to GIRK1/4.

In conclusion, by screening a total of 503 PF compounds, one compound and three compounds were identified as the most effective agonist and antagonists, respectively. These compounds were all selective for GIRK channels. These effective and GIRK-selective compounds may be useful, therefore, as possible therapeutics for drug dependence and pain.

## Figures and Tables

**Table 1:** IC_50_ Values of the Antagonists Selected in the Overall Screening

	PF40	PF236	PF246
GIRK1/2	6.06	5.98	24
(95%CI)	(4.03-8.68)	(3.09-10.3)	(13.4-55.6)
GIRK1/4	16.6	31.2	27.5
(95%CI)	(8.03-44.7)	(15.2-107)	(16.7-54.8)

CI: confidence intervals.
